# *Atypus karschi* Dönitz, 1887 (Araneae: Atypidae): An Asian purse-web spider established in Pennsylvania, USA

**DOI:** 10.1371/journal.pone.0261695

**Published:** 2022-07-07

**Authors:** Milan Řezáč, Steven Tessler, Petr Heneberg, Ivalú Macarena Ávila Herrera, Nela Gloríková, Martin Forman, Veronika Řezáčová, Jiří Král

**Affiliations:** 1 Crop Research Institute, Prague, Ruzyně, Czech Republic; 2 Tyler Arboretum, Media, PA, United States of America; 3 Third Faculty of Medicine, Charles University, Ruská, Prague, Czech Republic; 4 Faculty of Science, Laboratory of Arachnid Cytogenetics, Department of Genetics and Microbiology, Charles University, Viničná, Prague, Czech Republic; Sichuan University, CHINA

## Abstract

The mygalomorph spiders of the family Atypidae are among the most archaic spiders. The genus *Atypus* Latreille, 1804 occurs in Eurasia and northern Africa, with a single enigmatic species, *Atypus snetsingeri* Sarno, 1973, known only from a small area in southeastern Pennsylvania in eastern USA. A close relationship to European species could be assumed based on geographic proximity, but *A*. *snetsingeri* more closely resembled Asian species. This study was undertaken to learn more about the genetics of *A*. *snetsingeri*, its habitat requirements and natural history. Molecular markers (CO1 sequences) were compared to available data for other atypids and showed that *A*. *snetsingeri* is identical with *A*. *karschi* Dönitz, 1887 native to East Asia. Natural history parameters in Pennsylvania were also similar in every respect to *A*. *karschi* in Japan, therefore, we propose that the spider is an introduced species and the specific epithet *snetsingeri* is relegated to a junior synonym of *A*. *karschi*. Cytogenetic analysis showed an X0 sex chromosome system (42 chromosomes in females, 41 in males) and we also detected nucleolus organizing regions and heterochromatin, the latter for the first time in the Atypoidea. In Pennsylvania the spider is found in a variety of habitats, from forests to suburban shrubbery, where the above-ground webs are usually attached vertically to trees, shrubs, or walls, although other webs are oriented horizontally near the ground. Prey include millipedes, snails, woodlice, carabid beetles and earthworms. *Atypus karschi* is the first known case of an introduced purse-web spider. It is rarely noticed but well-established within its range in southeastern Pennsylvania.

## Introduction

Mygalomorph spiders of the family Atypidae are among the earliest divergent groups of spiders [1]. They dig a burrow and construct a ‘purse-web’, usually in the form of a closed tube, that occupies the burrow and extends above the ground horizontally or vertically for prey capture. The webs are well-camouflaged with soil particles and plant debris and potential prey are sensed when they walk on the surface of the tube. The spider impales the prey through the silk with its long fangs and injects paralytic venom. It then makes a slit in the tube large enough to drag the prey inside, repairs the tear with new silk, and feeds on the prey [2–5]. Atypid spiders spend their entire lifetime within their burrow in the silken web, 8–10 years for some females, and enlarge the burrow as they grow [6–8]. Males abandon their burrows when they reach maturity and wander in search of females, and then mate within the female’s web. Egg-laying occurs within the maternal web and fully capable spiderlings emerge later. In contrast to most mygalomorphs, atypid spiderlings utilize silk for aerial dispersal before establishing their first web [9–11]. This ability may have allowed some species of atypids to colonize new areas, including those that were uninhabitable during the last glacial period (e.g., northern Europe, [12], China [13: 38]).

There are currently three genera and 54 valid species of Atypidae [14]. The genus *Atypus* Latreille, 1804 (34 species from Europe, Asia, North Africa and North America), spins an above-ground web that is tubular and typically lays horizontally and parallel to the soil surface. In *Sphodros* Walckenaer, 1835 (seven species from eastern North America) the above-ground web is tubular and usually attached vertically to trees and other vegetation. In the genus *Calommata* Lucas, 1837 (13 species from Africa and Asia) the above-ground web is a flat circular pouch set on the soil surface [15].

The center of diversity of the genus *Atypus*, based on the number of species, is in southeastern Asia and at least three species live in the western Palearctic. Despite the number and widespread distribution of *Atypus* species they are secretive animals, and little is known about their habitat requirements, natural history, and genetic variation. In central Europe, certain *Atypus* species prefer sites with a microclimate regime resembling the climate of the glacial refuges from where they colonized the region [16]. The species that live on open steppe habitats require soils rich in calcium that maintain a favorable air humidity in spider burrows, and those that do not require calcic soils occur only in habitats sheltered by woody vegetation, and their webs are hidden in detritus [17]. As such, the European *Atypus* spiders are indicators of stable relic habitats and considered optimal flagship species in the conservation of disappearing relic xerothermic habitats [8].

Currently there are 16 species of Atypidae with available DNA sequence data, eleven of which represent the genus *Atypus* [18]. In contrast, only four atypid species, also in the genus *Atypus*, have been studied for their chromosomal constitution: *Atypus affinis* Eichwald, 1830; *Atypus karschi* Dönitz, 1887; *Atypus muralis* Bertkau, 1890; and *Atypus piceus*, Sulzer, 1776. The reported diploid number ranges from 14 to 44, and sex chromosome systems XY, X0, and X_1_X_2_0 have been described [19–21]. There are no data on other chromosome features, such as constitutive heterochromatin or nucleolus organizing regions (NORs). Those chromosome markers have been sporadically examined in Mygalomorphae [19,22].

This study looked at the genetics and habitat requirements of the lone species of *Atypus* found in North America, *Atypus snetsingeri* Sarno, 1973 [23]. This spider appears to be restricted to a small geographic area near Philadelphia, Pennsylvania in eastern USA [24]. It is morphologically very similar to *A*. *karschi* of Asia [7,25,26], and has been considered a possible introduction to North America. To help resolve its relationship with other atypids, the karyotype and genetic barcode (CO1) were developed for *A*. *snetsingeri* to compare with other *Atypus* species, along with observations on habitat associations and natural history. Data on karyotype [e.g., 27,28] and genetic barcode are frequently used to reconstruct phylogeny and evolutionary history of taxa including spiders.

## Material and methods

### Study locations

In November 2013 we visited eight sites in Delaware County, Pennsylvania, that were known to have *A*. *snetsingeri* populations [Tessler, personal observations]. The sites ranged from semi-urban areas near the type locality to wooded county parks along riparian corridors where purse-webs were common (S1 File). The primary site used for detailed web observations, specimen excavation and collection was a fallow field adjacent to forest at the Tyler Arboretum (hereafter "Tyler", Media, PA). That field was mowed annually to control invasive plants and facilitated access to the webs.

### Habitat and natural history

At each site, we assessed the primary vegetation cover and soil type. The land orientation of the web location was measured using a compass and the slope angles using an optical reading clinometer to the nearest 0.5°. Soil penetration resistance was measured as described by Srba & Heneberg [29], where higher values reflect mechanical impedance for burrowing.

The range of web sizes (tube diameter) was visually assessed in the field and prey were noted by identifying remnants of invertebrates found attached to webs. The webs of 18 adult females were excavated on 5–9 November 2013. The length of the purse-webs was measured, distinguishing the below-ground and above-ground sections by coloration and attached soil. The size of the females was characterized by measuring the length of the carapace along the midline. When juveniles were present, their number was counted. Several juvenile specimens were also excavated for the karyotype analysis (webs not measured). Voucher specimens from this study were deposited at the Crop Research Institute, Prague, Czechia.

### Statistical analysis

We used Pearson’s correlation test to analyze carapace size against web parameters (length of the below-ground and above-ground length) and to analyze the correlation between individual web parameters. We used Spearman’s rank correlation test to evaluate the correlation between female size and number of offspring. The difference in body size between females with offspring and females without offspring was analyzed using the Welch two sample *t*-test. We tested the variances in the below-ground and above-ground parts of the web by F-test. Normality was tested by the Shapiro-Wilk normality test. Data were analyzed in the statistical software R 3.6.2 [30]. The means are given with ± the standard error of the mean as a measure of sampling distribution.

### Karyotype analysis

Chromosome preparations were obtained from gonads of one immature male (testes present, sex can not be distinguished in living juveniles) and one mature female (ovary present). We followed the spreading technique described for mygalomorphs by Král et al. [22] except for fixation procedure. Due to a small size of gonads, we used two fixations (10 and 20 min) instead of three. The standard preparations were stained by 5% Giemsa solution in Sörensen phosphate buffer for 25 min. The evaluation of the karyotype was based on five mitotic metaphases. The chromosome measurements were carried out using ImageJ software [31]. The relative chromosome lengths were calculated in each specimen independently as a percentage of the total chromosome length (TCL) of the haploid set, including sex chromosome. Chromosome morphology was classified according to Levan et al. [32].

Our study also includes detection of constitutive heterochromatin and nucleolus organizing regions. Male mitotic plates were used to visualize these markers. Constitutive heterochromatin was detected by C-banding following Král et al. [33]. Preparations were stained by 5% Giemsa solution in Sörensen phosphate buffer for 75 min. NORs were visualized using fluorescence in situ hybridization (FISH) with a biotin-labeled probe for 18S rDNA sequences. The probe was obtained from *Dysdera erythrina* Walckenaer, 1802 (Dysderidae). FISH, probe detection by streptavidin-Cy3 and signal amplification was performed as described by Forman et al. [34].

### DNA extraction, amplification and sequencing

We isolated the DNA from legs of three *A*. *snetsingeri* individuals. We washed the ethanol-fixed legs twice for 15 min using 1 ml of 10 mM Tris-HCl (pH 7.5) with 5 mM EDTA. Subsequently, we extracted the DNA using a NucleoSpin Tissue XS kit (Macherey-Nagel, Düren, Germany) according to the manufacturer’s instructions. We then amplified the DNA using primers targeting nuclear (ITS2) and mitochondrial (CO1) loci using the following polymerase chain reaction mix: 10 mM Tris-HCl (pH 8.8), 50 mM KCl, 1.5 mM MgCl_2_, 0.1% Triton X-100, 0.2 mM dNTP (each), 1 μM forward primer, 1 μM reverse primer, 0.5 U of Taq DNA polymerase (Top-Bio, Prague, Czech Republic), and 300 ng of extracted genomic DNA. The total reaction volume was 25 μl. To amplify the ITS2 locus, we used the primers ApicITS2FW2 (5′-CGATGAAGAACGCAGCCAGCTGCGAG-3′; [35]) and RITS (5′-TCCTCCGCTTATTGATATGC-3′; [36]). To amplify the CO1 locus, we used the primers LCO1490 (5′-GGTCAACAAATCATAAAGATATTGG-3′; [37] and C1-N-2194 (5′-CTTCTGGATGACCAAAAAATC-3′; [38]). We performed the reaction using an Eppendorf Mastercycler Pro thermal cycler (Eppendorf, Hamburg, Germany) for 36 cycles with 15-s denaturation at 94°C, 2-min annealing at 43–57°C, followed by a 1–3-min extension at 72°C. We initiated the cycling with a 2-min denaturation at 94°C and terminated it after 5-min incubation at 72°C. Subsequently, we purified the amplified DNA using USB Exo-SAP-IT (Affymetrix, Santa Clara, CA) and bidirectionally sequenced the amplicons using an ABI 3130 DNA Analyzer (Applied Biosystems, Foster City, CA). For the three individuals of *A*. *snetsingeri* analyzed in their ITS2 locus and two for their CO1 locus, all the obtained ITS2 and CO1 sequences were identical. The resulting consensus DNA sequences were submitted to NCBI GenBank under accession numbers MT957000-MT957001 (CO1) and MT957146- MT957148 (ITS2).

### Alignments and phylogenetic analyses

We aligned the newly generated sequences with those of nine *Atypus* spp. obtained from NCBI GenBank as of September 7, 2020, and sequences of the corresponding outgroups by using MUSCLE [39,40] (gap opening penalty -400, gap extension penalty 0, clustering method UPGMB, lambda 24). We manually corrected the alignments for any inconsistencies, trimmed the aligned sequences to ensure that they all represent the same extent of the analyzed locus, removed short-length sequences from the alignments, and used only trimmed sequences for further analyses. The trimmed ITS2 locus [containing partial 5.8S ribosomal DNA and partial (close to full-length) ITS2 sequences] corresponded to nt 62–385 (324 bp) of *Atypus baotianmanensis* Hu, 1994 KP208877.1. The trimmed CO1 locus (partial CO1 coding sequence) corresponded to nt 23–595 (573 bp) of *A*. *piceus* KX536935.1. For each locus, we calculated the maximum likelihood fits of 24 nucleotide substitution models. We used a bootstrap procedure at 1,000 replicates and the nearest-neighbor-interchange as the maximum likelihood heuristic method to determine the tree inference when the initial tree was formed using a neighbor joining algorithm. We used best-fit models for the maximum likelihood phylogenetic analyses, including the estimates of evolutionary divergence between sequences.

### Ethics statement

We studied spiders that are not protected by any law, and specimens were collected ethically with permission of the land owner. Approvals for such studies are not required from ethics committees in either the USA or the Czech Republic.

## Results

### Phylogenetic analysis

**Fig 1:**
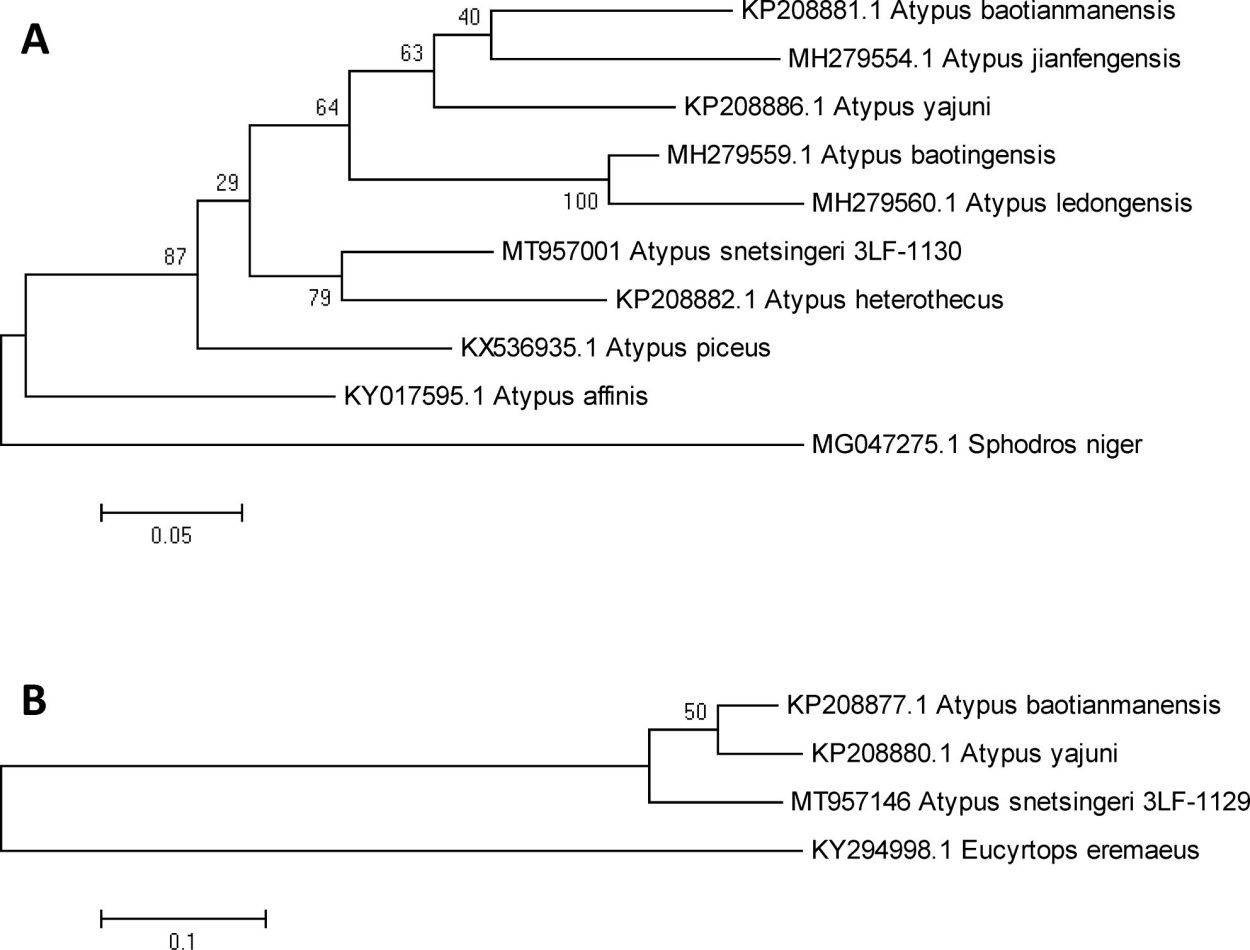
Phylogenetic analyses of the position of *A*. *snetsingeri (A*. *karschi* in Pennsylvania, USA) in the genus *Atypus* based on the sequences of the CO1 (**A**) and ITS2 (**B**) loci by the maximum likelihood approach. The evolutionary history was inferred using the Tamura-Nei model (**A**) or the Kimura 2-parameter model (**B**), both with a discrete Gamma distribution used to model evolutionary rate differences among sites. The models were selected based on the highest Bayesian information criterion scores of the maximum likelihood fits. The trees are drawn to scale, with branch lengths indicating the number of substitutions per site. All codon positions, including noncoding positions, were included; the analyses were based on 573 positions (**A**) or 345 positions (**B**).

Sequence analysis of the DNA of *A*. *snetsingeri* has clarified its identity and the unusual presence of the genus in North America. We found that the CO1 locus had a 100% sequence similarity (genetic distance of zero) with the matching 639bp-long CO1 locus of *A*. *karschi* (SDSU_MY4706) from the Honshu Island, Japan [41]. After *A*. *karschi* the most closely related species for which sequences were available (Fig 1A) was the Asian *Atypus heterothecus* Zhang, 1985, with a genetic distance of 0.131 ± 0.021 of base substitutions per site between sequences. The European species, *A*. *piceus* and *A*. *affinis*, were basal to *A*. *snetsingeri* as well as to the whole group of hitherto sequenced Asian *Atypus* spp. (Fig 1A). Concerning the ITS2 locus, the sequences of only two other *Atypus* spp. are known (Fig 1B); therefore, this hypervariable locus awaits future analyses when more comparative data are available. The genetic distance to the closest species already sequenced in the ITS2 locus, *A*. *baotianmanensis*, was 0.109 ± 0.022 of base substitutions per site between the sequences.

### Taxonomy

Based on an exact match of the genetic CO1 barcode data, the *A*. *snetsingeri* purse-web spiders in Pennsylvania appear to represent an introduced local population of the Asian species *A*. *karschi*. In the remainder of this paper those spiders are referred to as *A*. *karschi ‘*from Pennsylvania’. The specific epithet *snetsingeri* is relegated to a junior synonym of *karschi*.

***Atypus karschi*** Dönitz, 1887

*Atypus snetsingeri* Sarno, 1973: Sarno 1973 [23]: page 38, figs 1–9 (description of both sexes). **New synonymy.**

*A*. *snetsingeri* Gertsch and Platnick 1980 [25]: page 11, figs 9, 13–20 (both sexes).

*A*. *snetsingeri* Schwendinger 1990 [7]: page 360, fig. 18 (female).

#### Remarks

The synonymy was based on finding that the CO1 gene, used as a molecular barcode, of *snetsingeri* specimens from Pennsylvania was identical with that of *A*. *karschi* from the Honshu Island, Japan [41].

### Cytogenetic analysis

**Fig 2:**
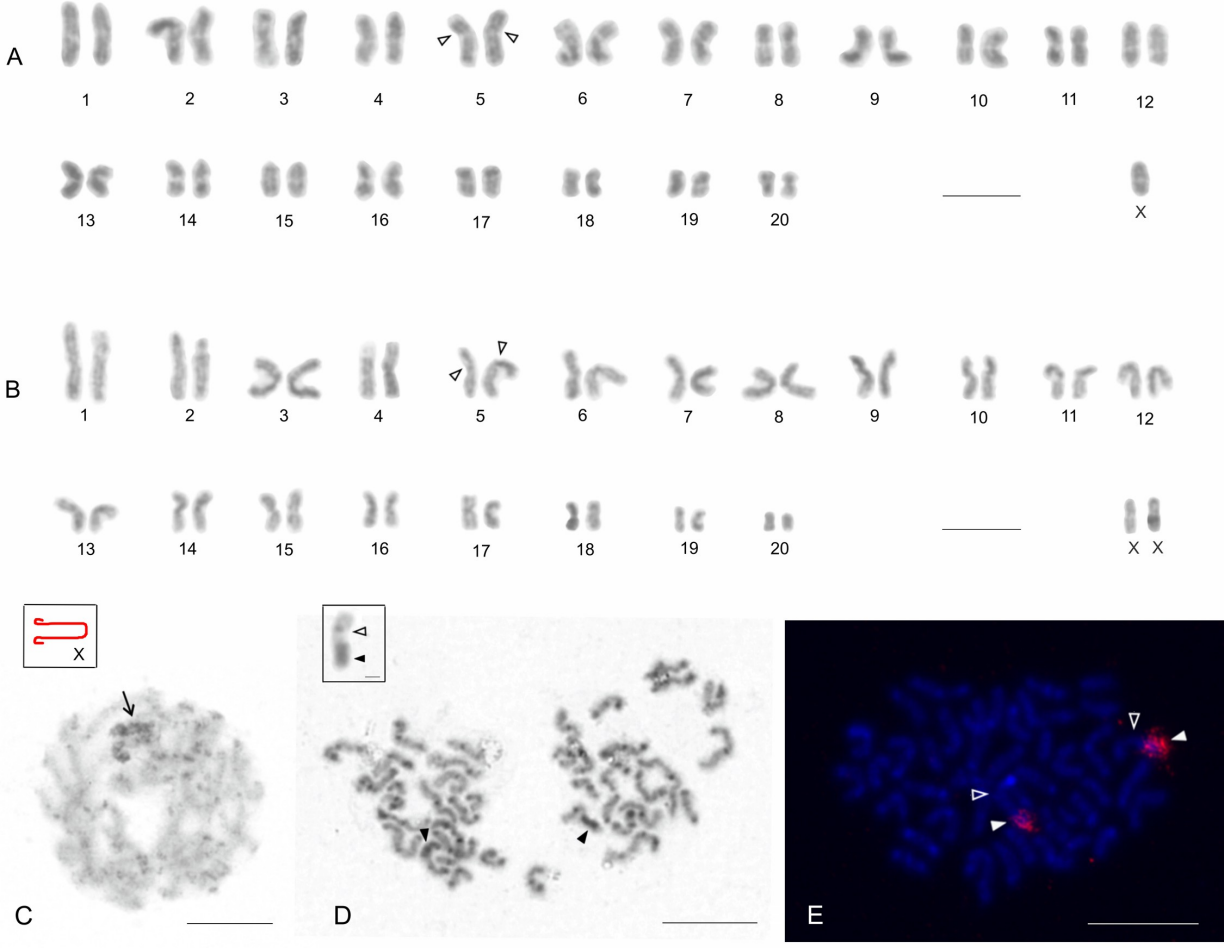
Chromosomes of *A*. *karschi*, Pennsylvania, USA. **A, B.** Male (A) and female karyotypes (B), stained by Giemsa, based on mitotic metaphase. 2n♂ = 41, X0; 2n♀ = 42, XX. Empty arrowhead–centromere of submetacentric pair. **C.** Male pachytene. Note heterochromatic X chromosome on the periphery of the nucleus and prominent knobs on the bivalents. Inset: Scheme of sex chromosome. Note an association of X chromosome arms. Arrow–sex chromosome. **D.** Male mitotic metaphase, C-banding. Chromosomes exhibit intercalar and terminal heterochromatin blocks. Inset: Magnified submetacentric chromosome containing a large block of heterochromatin (from another mitotic metaphase). Arrowhead–a large block of heterochromatin, empty arrowhead–centromere. **E.** Male mitotic metaphase, detection of rDNA cluster (FISH). Note chromosomes of a submetacentric pair containing a terminal rDNA cluster at long arm. Arrowhead–rDNA cluster, empty arrowhead–centromere. Scale bars: 10 μm.

The female karyotype of *A*. *karschi* from Pennsylvania showed 2n = 42 chromosomes and the male had 2n = 41 (Fig 2A), suggesting an X0 sex chromosome system. Chromosomes were metacentric except for one pair, which exhibited submetacentric morphology (Fig 2B). The chromosome pairs gradually decreased in size, with the length of chromosome pairs in the male ranging from 7.13% to 3.31% of TCL and in the female from 6.13% to 3.31% of TCL. The sex chromosome was a metacentric element of medium size in both male (TCL = 4.27%) and female (TCL = 4.09%) (Fig 2A and 2B). Concerning meiosis, pachytene nuclei were found in both the male and female specimen. In the male pachytene, the univalent X chromosome was on the periphery of the nuclei. X chromosome arms were often associated with each other during this period. Moreover, the X chromosome showed positive heteropycnosis (i.e., it was stained more intensively than other chromosomes). The other bivalents exhibited prominent knobs (Fig 2C).

C-banded chromosomes exhibited small intercalar and terminal blocks of heterochromatin. The submetacentric pair showed a prominent large block at the terminal part of the long arm (Fig 2D). It occupied on average 36% of the chromosome length (n = 10). The karyotype contained one NOR locus that was localized in the end of the long arm of the submetacentric pair (Fig 2E). The NOR colocalised with the large block of heterochromatin and was of considerable size (37.2% of the chromosome length, n = 8).

### Habitat

**Fig 3:**
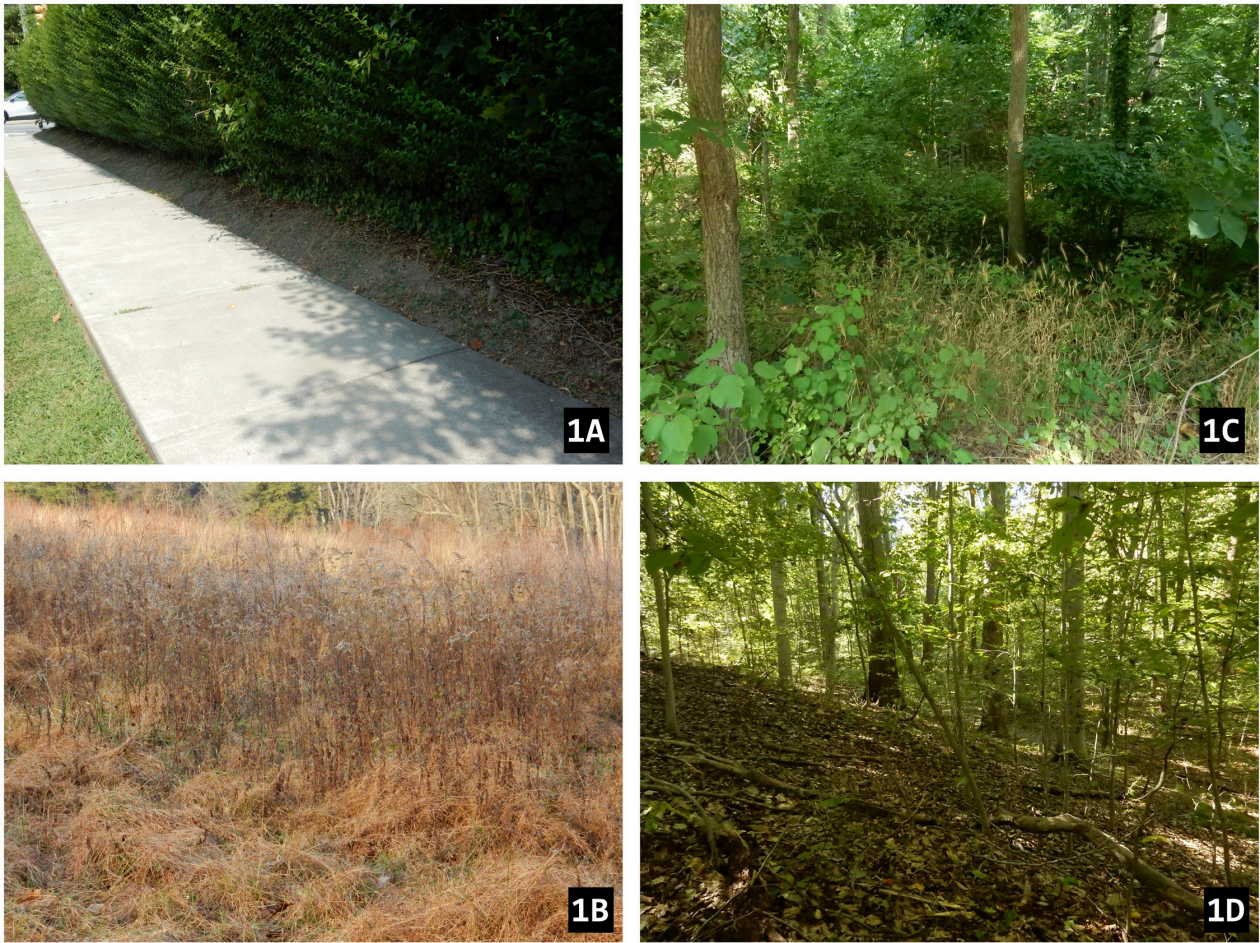
Habitats of *A*. *karschi* in Pennsylvania, USA; (1A) suburban bushes along Essex Ave (~200 m from type locality of *A*. *snetsingeri*), (1B) fallow field at Tyler Arboretum, (1C) riparian woods, Swedish Cabin site on Darby Creek, (1D) forest, Smedley Park.

The eight *A*. *karschi* sites that we visited in Delaware County in 2013 represented suburban neighborhoods, small wooded parks, narrow riparian zones along developed stream corridors, and protected parklands (S1 File). The purse-webs were located in a variety of habitats at those sites, including the shrubbery along suburban sidewalks, slopes and bottoms of wooded valleys, beech forests and a fallow field that is mowed annually. Typical habitats of *A*. *karschi* in Pennsylvania are shown in Fig 3.

The inclination (slope) of the sites varied from 0–40°, ranging from a flat field to riparian hillsides. Where a site in our study had a slope it usually faced the south but the azimuth of orientation varied from 95–340°, excluding only the coldest north and north-east exposures. The soil on slopes was usually not aggregated, was sandy or powdery, and of yellow or grey color below the shallow humus layer. In valley bottoms, the spider lived in fluvisol and in the suburbs in anthropogenic soils. Soil penetrability ranges from 0.5 to 3.25 (n = 14, mean 2.02 ± 0.31). The webs were typically associated with woody vegetation, and bush/shrub cover ranged from 5–100% (mean 40%) and tree cover from 0–90% (mean 50%). The soil surface where webs occurred was without moss, and the herbaceous cover was usually sparse (from 0–90%, mean 20%).

### Natural history

**Fig 4:**
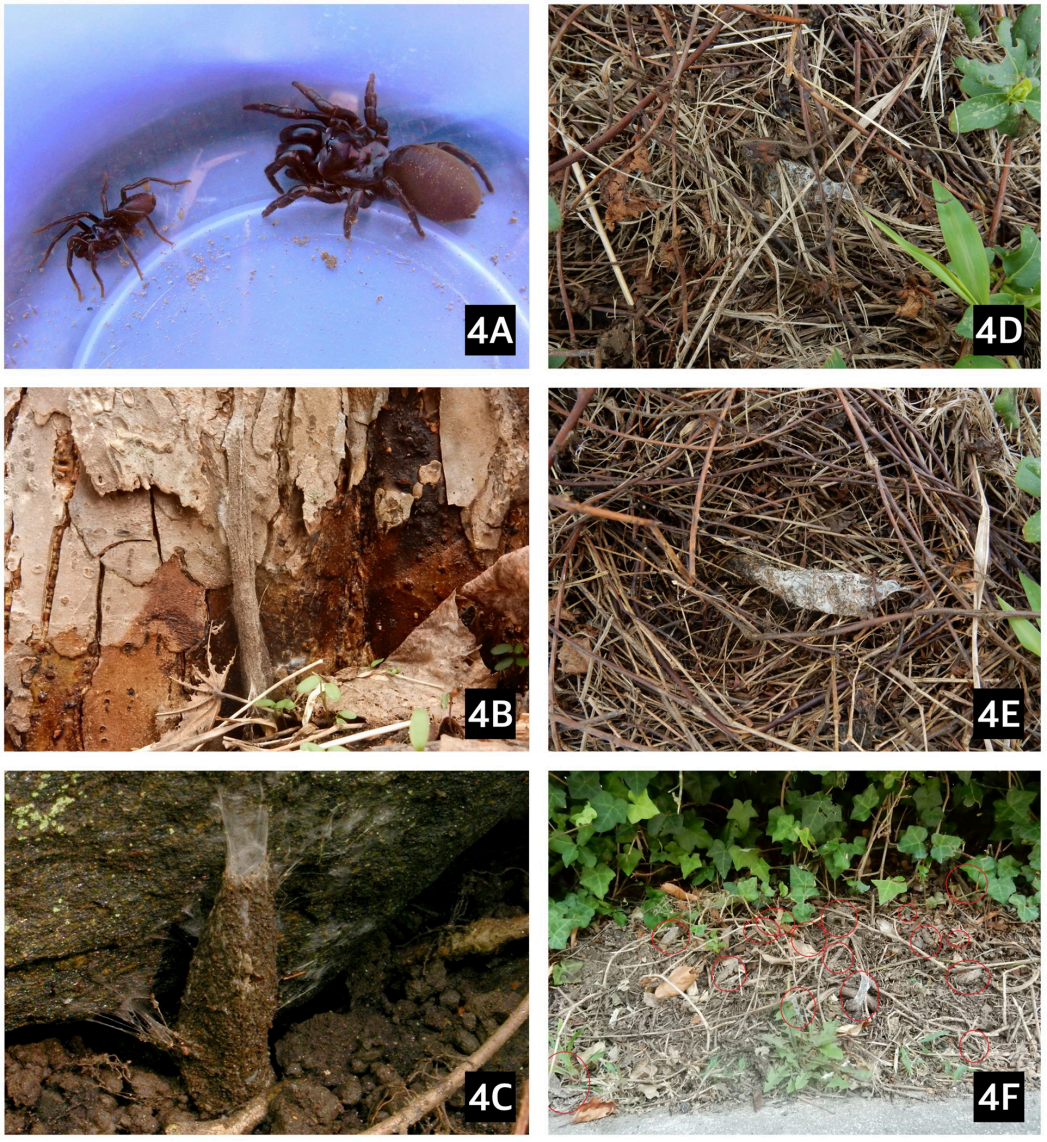
*A*. *karschi* and its webs in Pennsylvania, USA, (4A) adult female and male (on the left), (2B) vertical web attached to the base of a tree and (4C) to a boulder, (4D) horizontal web covered in thatch (4E) and with thatch removed, (4F) a detail of recently trimmed ivy in front of the same bushes shown in Fig 3A, with 16 purse-webs circled.

The above-ground webs we observed were vertical and mostly attached to the base of thin stems of bushes or on trees (Fig 4B), but a few were attached to rock (Fig 4C). In early November three size categories were visually distinguished in the field by their relative web diameters, representing small and medium juveniles, and adult females. According to prey remnants found on their webs, they feed on millipedes (*Julida* and *Polydesmus* sp.), snails (*Cochlicopa* sp.), woodlice (*Porcellio* sp.) and carabid beetles.

**Fig 5:**
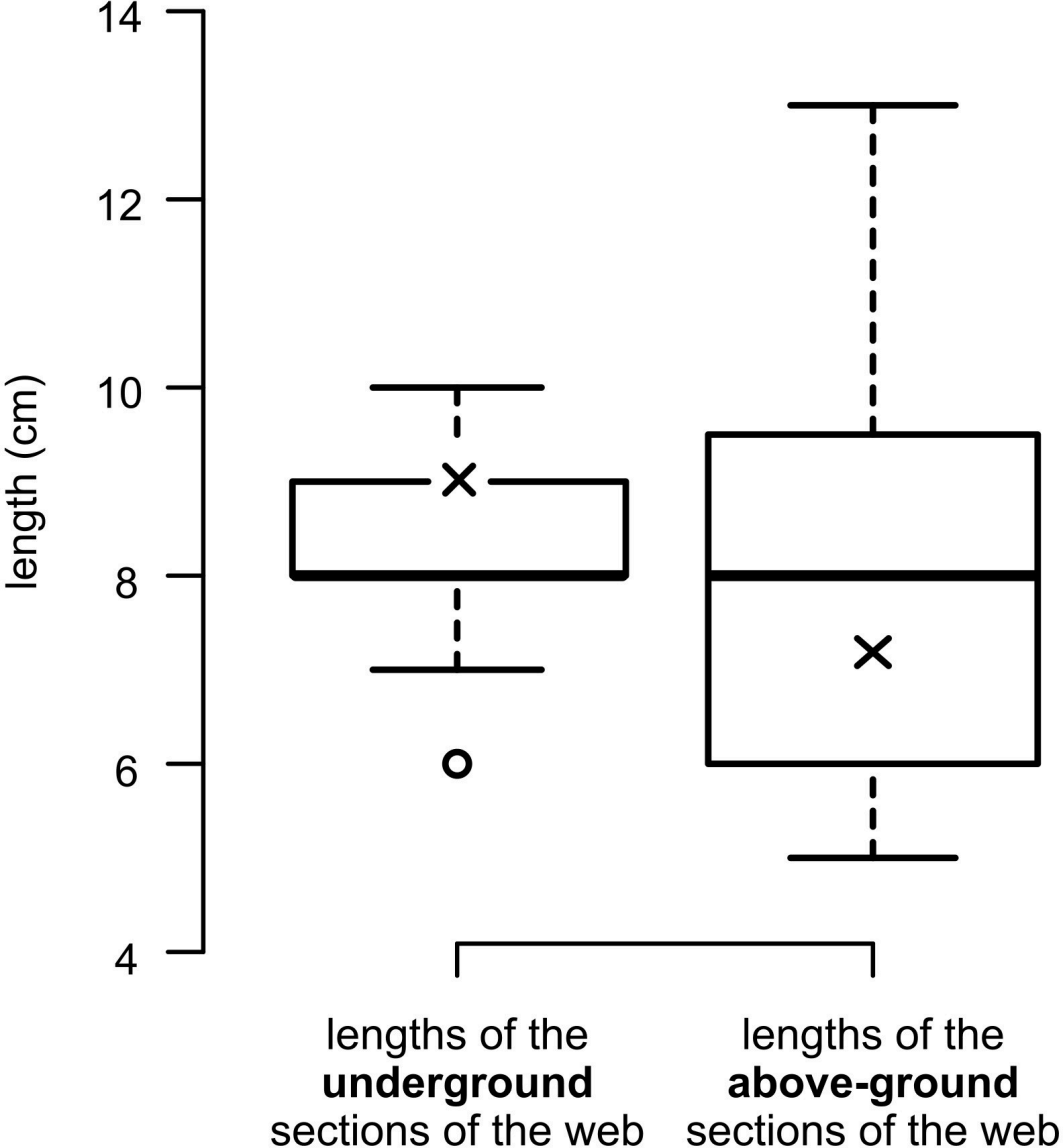
Boxplots showing the variation of the below-ground and above-ground lengths of excavated purse-webs of *A*. *karschi*, Pennsylvania, USA (n = 13). The means are indicated by an x and the hollow dot indicates an outlier (less than the 25th percentile minus 1.5 × Interquartile range).

All 18 excavated webs contained adult females (S2 File), although some webs were damaged in the process. Eleven webs also contained a brood of juveniles, one of which was partially lost during excavation and not used for count statistics. Four of the webs were badly damaged during excavation and were not measured. Of the remaining 14 webs, one had a broken below-ground section that was only partially intact and not used for statistics. Body size of the females (carapace length) varied by only about 1mm (n = 18, min 5.04 mm, max 6.18 mm, mean 5.68 ± 0.09 mm). There was no significant difference between the body size (carapace length) of the females with (n = 11) and without (n = 7) juveniles (Welch two sample *t*-test t = 1.45, *p* = 0.17). The number of juveniles ranged from 70 to 201 (n = 10, mean 121.30 ± 11.66) and did not correlate with the body size of the female (Pearson’s correlation n = 10, r = 0.32, *p* = 0.37). Total web length (both sections intact) ranged from 13 to 29 cm (n = 13, mean 17.77 ± 1.13 cm). The length of the subterranean section of web associated with the burrow ranged from 6 to 10 cm (n = 13, mean 8.3 ± 0.3 cm) and did not correlate with the body size of the spider (Pearson’s correlation, n = 13, r = -0.44, *p* = 0.13). The length of the above-ground web ranged from 5 to 13 cm (n = 14, mean 8.54 ± 0.67 cm) and also did not correlate with the body size of the spider (Pearson’s correlation, r = 0.02, *p* = 0.94). The length of the above-ground web was more variable than the length of its subterranean part (F test, n = 13, F = 0.23, *p* = 0.018) (Fig 5). The ratio of below-ground/above-ground lengths ranged from 0.62 to 1.80 (n = 14, mean 1.09 ± 0.08) and did not correlate with the body size of the spider (Pearson’s correlation, r = 0.02, *p* = 0.94).

## Discussion

### Genetic identity of *A*. *snetsingeri*

The presence of a geographically isolated population of an *Atypus* species in North America, where the other purse-web spiders are in the genus *Sphodros*, has been mildly controversial. Due to the species’ location on the eastern coast of the USA a close relationship with European *Atypus* species could have been expected. However, morphologically, *A*. *snetsingeri* closely resembled Asian species, especially *A*. *karschi* [7,25]. Raven [26] questioned whether the *Atypus* in North America was introduced.

The newly obtained molecular data for *A*. *snetsingeri* have resolved those questions by showing that the Pennsylvania species is conspecific with Asian *A*. *karschi*. Sequence data of *A*. *snetsingeri* was a genetic match with sequence data for *A*. *karschi* from Japan, supporting an East Asian origin and introduction. Based on these data we propose a formal synonymy for *A*. *snetsingeri*, which now becomes a junior synonym of *Atypus karschi*. Differences reported for morphological features compared to *A*. *karschi* in Asia probably represent intraspecific variation given the small number of *A*. *snetsingeri* specimens actually examined by researchers [7,25].

Parts of the genome of “*Atypus snetsingeri”* (based on NCBI sequences DQ639853.1, DQ680323.1 and KY016940.1) were previously used in spider phylogeny studies to represent the genus *Atypus* [41–43] or the entire family Atypidae [44]. Wheeler et al. [1] used *A*. *snetsingeri* and *A*. *affinis* data to represent *Atypus*, and added *Sphodros* for the family Atypidae. Those *A*. *snetsingeri* data are now considered to represent *A*. *karschi* in Pennsylvania. Recently the entire mitochondrial genome was sequenced for *Atypus karschi* in China [45], which is very useful for further comparative studies of the Atypoidea.

### Cytogenetic analysis

Most karyotype data on spiders concerns araneomorphs [46], but some karyotypes of mygalomorph spiders have been published [19,20,22,47,48]. Representatives of the superfamily Atypoidea display a similar range of diploid numbers as araneomorph spiders (from 14 to 47). Most Atypoidea exhibit the X0 sex chromosome determination system, which may be the ancestral sex chromosome determination of this superfamily [22].

In the family Atypidae only four species in the genus *Atypus* have been studied cytogenetically. *Atypus karschi* in this study exhibits 2n♂ = 41, X0 and predominance of metacentric chromosomes, which is in accordance with the karyotypes of central European species *A*. *piceus* and *A*. *muralis* [20]. These karyotype features could be ancestral within the genus *Atypus*. The karyotype of European *A*. *affinis* having 2n♂ = 14, XY, was derived from chromosomal complement 2n♂ = 41, X0 by series of chromosomal fusions leading to decreasing of diploid count and formation of a neo-sex chromosome system XY [20].

Notably, an earlier karyotype developed for *A*. *karschi* in Japan [21] differs considerably from those reported in this study from Pennsylvania. The male karyotype reported from Japan consisted of approximately 44 acrocentric chromosomes, including an X_1_X_2_0 system, not the 41 biarmed (i.e. metacentric and submetacentric) chromosomes and X0 pattern reported here. The discrepancy may be due to interpopulation variability, but although mygalomorph spiders exhibits considerable karyotype diversity [22], such an enormous degree of interpopulation karyotype variability is very unlikely. Therefore, we suggest that the karyotype data of the Japanese population may have been misinterpreted. The karyotype of *Atypus* is formed by a relatively high number of small chromosomes, which makes it difficult to determine the precise diploid number and chromosome morphology. Moreover, the method of chromosome preparation used by Suzuki [21] did not include treatment with a hypotonic solution, so the spreading of chromosomes would have been less pronounced than in the present study using the methodology of Král et al. [22]. Regarding determination of the sex chromosome system, a single metacentric X chromosome of an X0 system could be erroneously considered as two acrocentric X chromosomes of an X_1_X_2_0 system attached at one end during meiosis.

Within the framework of our cytogenetic analysis, we were able to detect constitutive heterochromatin for the first time in the Atypoidea. Most chromosomes of *A*. *karschi* exhibited intercalar and terminal blocks of heterochromatin. The distribution of blocks suggests that 1) most intercalar blocks are placed at centromeric regions and 2) terminal blocks are formed at telomeric regions. This is consistent with the pattern of distribution of constitutive heterochromatin most commonly found in spiders [47].

Nucleolus organizer regions are chromosome domains containing tandemly repeated sequences of rRNA genes that are involved in formation of the nucleolus [49], and their location on chromosomes may have taxonomic value. These regions have been detected in ten species of mygalomorphs including four species of Atypoidea [22, this study]. The number of NORs in Atypoidea ranges from one to four loci, and they are always situated on chromosome pairs. NORs are usually detected by impregnation with silver or by FISH with rDNA probe, although the first technique can underestimate absolute number of NORs by visualizing only loci transcribed during previous cell cycle [50]. However, most NOR detections in mygalomorphs have been performed by silver staining. Fluorescence in situ hybridization, which we applied to detect NORs of *A*. *karschi* in this study, have been used with only one other mygalomorph species, *Tliltocatl albopilosum* Valerio, 1980 (Theraphosidae) [22]. Both species display one terminal NOR localized on a chromosome pair, which may be the ancestral condition for spiders [22]. The NOR of *A*. *karschi* is associated with heterochromatin, which is a common feature of rDNA in eukaryotes [e.g., 51,52]. Comparison of the length of the rDNA cluster and heterochromatin block suggests that heterochromatin associated with the NOR is formed by inactivated rDNA. This pattern is in an agreement with the current model for NOR organization, in which major regions of rDNA are often inactivated and only a restricted fraction of rDNA is transcribed [53].

### Habitat

Asian *Atypus* species are generally considered to be forest-dwellers, but some are reported from more open xerothermic habitats [7,62]. Details about natural history for most species is limited to brief remarks in the taxonomic literature, but those comments can include important observations about capture method, habitat features or tree species associated with the spiders or their webs at the time of collection [7,13,62]. These types of data increase in value over time for planning future surveys and tracking spider distributions or habitat changes over time.

*Atypus karschi* in Pennsylvania appears to be undemanding regarding habitat requirements (see the S1 File) and can be locally abundant where it occurs. In Pennsylvania this species is found in protected riparian woodlands, brushy woodlots and at least one mowed field, and is also reliably found in some suburban neighborhoods where webs are built at the base of shrubs or along walls and fences. Miyashita [54] reported a similar situation in Japan where *A*. *karschi* is “common” and “usually live(s) in shady and humid places such as woods and shrubberies.” Images posted on iNaturalist [55] of *A*. *karschi* in East Asia also support a tolerance of human-modified settings where they were encountered (wall, fence and stone garden).

Unlike *A*. *karschi* in Pennsylvania, the three European *Atypus* species are not found in habitats subjected to recent or regular disturbance, and they are well studied. They are known to require habitats with specific edaphic conditions and association with particular vegetation types and sun-facing slopes [16]. European atypids are rare enough to be red-listed in all Central European countries, and their presence at a site is an indicator of a relic habitat worthy of conservation management [8,56].

### Natural history

The life history of *A*. *karschi* in Japan was studied in detail by Miyashita [54] under semi-outdoor conditions and reported with prior data from Aoki [57] and Yaginuma [58]. Basic natural history parameters of *A*. *karschi* in eastern Asia and *A*. *snetsingeri* in the USA are contrasted in Table 1 and indicated a similarity in every respect (body size, ontogeny, phenology, fecundity, morphology of webs, environment). No difference was found that would refute the conspecificity of the Pennsylvania population with Asian *A*. *karschi*.

**Table 1:** Natural history parameters (body size, ontogeny, phenology, fecundity, morphology of webs, environment) reported for *Atypus karschi* in Japan and for the introduced population known as *A*. *snetsingeri* in Pennsylvania, USA.

Natural history parameter	*Atypus karschi* (Japan)	*Atypus snetsingeri* (USA)
**Body size**		
Carapace length of males	3.87–4.23 mm [59]	3.2–4.6 mm [23]
Carapace length of females	4.77–5.76 mm [59]	3.4–7.0 mm [23]
**Ontogeny**		
No. of eggs	mean 124, maximum 270 [54]	mean 121, maximum 201 (this study)
No. of moults before reaching maturity	8–9 in males, 9–11 in females [54]	unknown
Age of maturation	3 years [54]	possibly 3 years, based on three concurrent web size categories in the population (this study)
**Phenology**		
Mating season	June–(July) August [54,57]	June–August [23,24]
eggs	July (August)—September [54,57,58]	July-September [24]
larvae	October [60,61]	September [24]
1st nymphal instar	late October–April (dispersion) [54,57]	September–March (dispersion) [24]
**Morphology of webs**		
Orientation of the above-ground web	vertically attached to the tree trunk or rock [61]	vertically attached to the tree, hedge or wall [23] or horizontally oriented in grass and thatch [24]
Length of the above-ground web	Up to 20 cm (almost the same as the depth of the burrow) [61]	Up to 25 cm [23]
Depth of the burrow	Up to 20 cm [54,61]	Up to 20 cm [23]
**Environment**		
Microclimate	Shady and moist, in the forest close to the trees, rocks or bamboo [54,61]	Mostly shady and moist, in litter and areas with loose soil [this study]
Habitat	Forests and shrubs [54,61]	Forests and shrubs, disturbed areas [this study]

The webs of *A*. *karschi* in Pennsylvania are attached to a variety of supports (trees, shrubs, grasses, rocks, walls and fences) where the ground surface is either covered by or nearly devoid of litter. Gertsch and Platnick [25] contemplated whether the above-ground purse-web orientation could be useful to distinguish between *Atypus* (horizontal webs) and *Sphodros* (vertical webs), but this is not a distinguishing character as species in both genera can and do make both kinds of webs [62]. In this study in 2013 we observed vertical webs of *A*. *karschi* at the sites visited, but the spiders are also known to make horizontal webs in thatch and grass [24]. In Tyler’s fallow field, for example, vertical webs can be found on plant stems within a few centimeters of horizontal webs in surrounding grasses.

Although vertical webs are characteristic of most North American *Sphodros* species, *Sphodros niger* Hentz, 1842 may preferentially build horizontal webs, at least in some settings [63,64]. Mckenna-Foster et al. [65] also found that *Sphodros rufipes* Latreille, 1829 in New England used whatever support was available and many webs were close to the ground. The suggestion that horizontal webs are an adaptation to prey capture under the snow [7] may ignore the function of vertical webs at ground level. In Pennsylvania *A*. *karschi* habitats experience snow and cold temperatures each year. In Tyler’s field the horizontal webs laying near the soil surface tend to be well-buffered by leaf litter or thatch, but basal sections of nearby vertical webs are often similarly buffered and may likewise function normally in the same subnivean environment when both prey and spiders are active [Tessler, personal observations].

In this study we measured the webs of fourteen adult females from a fallow field with homogeneous soil. We found the overall length of the webs were shorter than those observed by Sarno [23] around a house foundation and on shrubs in a suburb (see Table 1), probably reflecting different conditions and prey availability between sites. The length of the aerial web was more variable than that of the underground part (Fig 5). Less variation in the underground web length may reflect a minimum depth of the burrow necessary for suitable microclimate, constraints imposed on digging, or the shallow soil frost depth in winter at that site. Depth of burrows differs among European *Atypus* species, where the species living in arid habitats tend to dig deeper burrows than those living in woody vegetation [8].

The number of juveniles found within maternal webs of *A*. *karschi* in Pennsylvania and Asia were similar (max. 201 and 270, respectively), and in the same range as European *Atypus* species (*A*. *affinis* max. 191, *A*. *piceus* max. 168, *A*. *muralis* max. 150; M. Řezáč, personal observations). Mortality of captive *A*. *karschi* spiderlings is reportedly high [23,56]. In mygalomorphs, spiderlings disperse by walking away from the maternal web, sometimes in single file, or by using a dragline-based aerial method known as suspended ballooning [11]. The extent to which either dispersal behavior contributes to spiderling mortality is unknown. In Pennsylvania, aerial dispersal occurs in March and April for *A*. *karschi* [24] in both field and forest habitats.

Prey we observed for *A*. *karschi* in Pennsylvania were mostly ground-based invertebrates, especially millipedes, similar to observations on *S*. *niger* in New England [64]. *A*. *karschi* in Pennsylvania has also been observed feeding on earthworms, and will readily capture orthopteroids and other insects that contact the web while climbing vegetation, including the pestiferous spotted lanternfly (Hemiptera: Fulgoridae: *Lycorma delicatula* White, 1845) that was recently introduced into Pennsylvania from Asia [Tessler, personal observations]. It is unknown what effect *A*. *karschi* has on its local environment or prey species.

### Distribution of *A*. *karschi* in Pennsylvania

Prior to this study, *A*. *snetsingeri* had been considered a unique species with a decidedly Asian morphology and an enigmatic presence in northeastern North America, near Philadelphia. Raven [26: pg 124] used a parenthetical remark to question whether the isolated *Atypus* in North America was "[possibly introduced]," and Schwendinger [7: pg 364] agreed it could explain *A*. *snetsingeri*’s "restricted distribution in a generally well investigated area." Schwendinger [7] also recognized that the "obviously close" relationship of the species pair *A*. *karschi/A*. *snetsingeri* resembled the well known biogeographic pattern referred to as the eastern Asian—eastern North American disjunction [66,67], one of several intercontinental disjunctions of interest in paleontology and systematics. Zhu et al. [13: pg 38] agreed that the species may have spread southward during the Pleistocene, but rejected Schwendinger’s land bridge hypothesis and "would rather consider *A*. *snetsingeri* as a relict of glacial periods."

Based on current information, we disagree with both natural origin scenarios for the occurrence of *A*. *karschi* in North America and believe that the Pennsylvania population is the result of introduction by man and not ancient vicariance. Mygalomorph spiders usually have deep molecular differences even among populations of the same species [41]. But *A*. *snetsingeri* is genetically identical to *A*. *karschi*, and its distribution and association with disturbed habitats suggest a more recent introduction.

*A*. *karschi* seems to possess three preadaptations that allowed it to successfully colonize southeastern Pennsylvania following its introduction. First, it occurs over a wide area in eastern Asia with a similar temperate climate (Japan [61]; Chinese provinces Hebei, Anhui, Sichuan, Guizhou, Hubei, Hunan, Fujian [59]; Taiwan [68]; Korea’s Ungil Mountain [69]). Second, it produces a large number of lightweight juveniles that disperse aerially using a method known as suspended ballooning [11,24,54,70]. Third, our results in Pennsylvania indicate that the species is ecologically plastic and does not appear to have specific habitat, edaphic or microclimatic requirements, even thriving in settings frequently impacted by humans.

**Fig 6:**
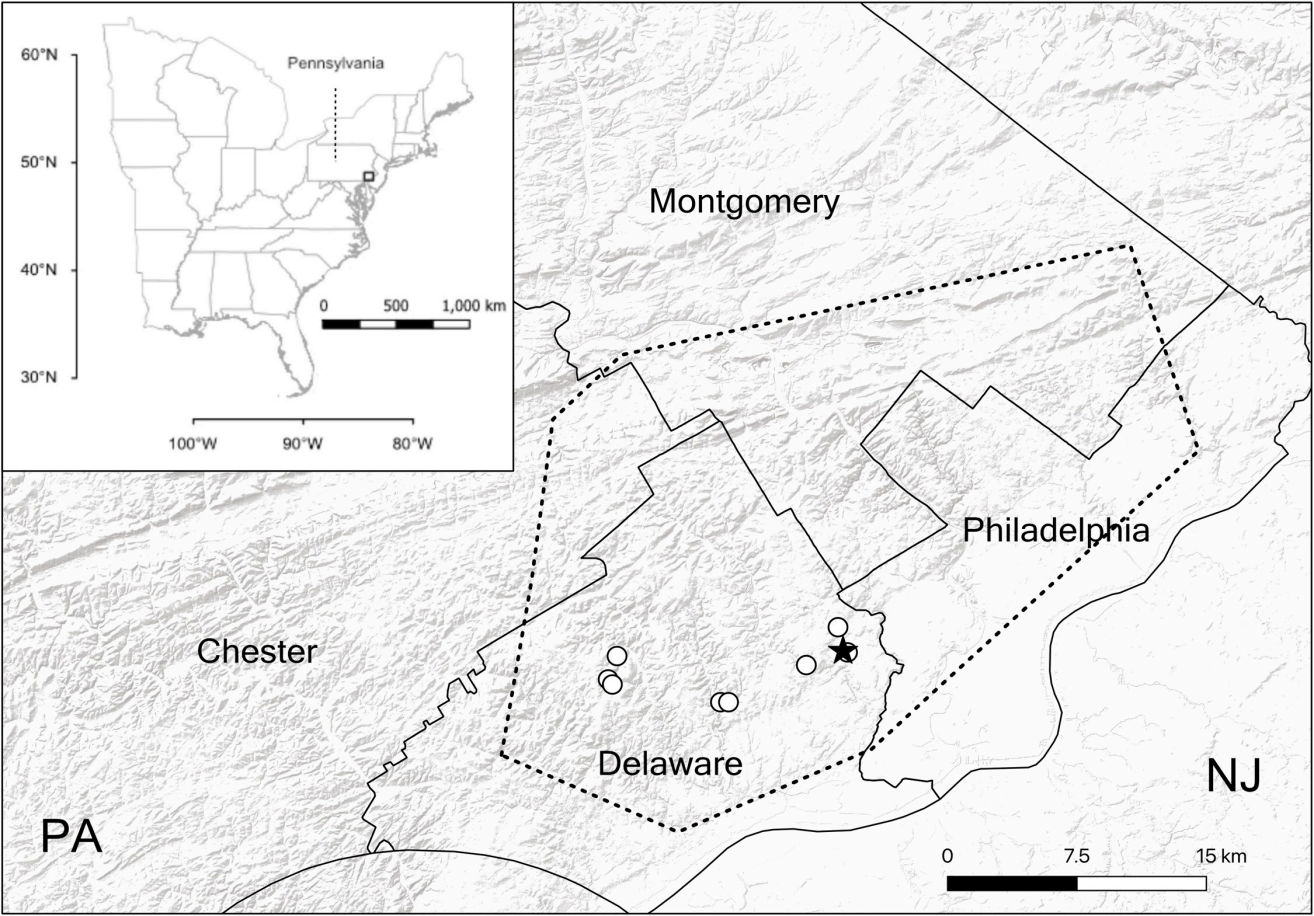
Study sites, counties and estimated area containing *Atypus karschi* in southeastern Pennsylvania, USA. The hollow circles mark the sites described in this study and the star is the type locality of *A*. *snetsingeri*. The minimum convex polygon encloses a nearly contiguous area where vertical purse-webs have been found in a recent visual survey in the region [24], and includes the relatively few sites where a species identification to *A*. *karschi* (as *A*. *snetsingeri*) has been made through previous collections, male sightings and female excavation and examination. *Sphodros* species may also be found in that area as purse-web sites are revisited for species determination.

The original description and first review of the species *A*. *snetsingeri* in Pennsylvania were based on specimens taken from two nearby suburban sites in Lansdowne and Upper Darby in eastern Delaware County near Philadelphia [23,25]. At that time, the spider was known to be common and unnoticed in the surrounding areas within the Cobbs Creek and Darby Creek drainage basins [Tessler, personal observations]. Since that time a visual survey for vertical purse-webs at publicly-accessible parks and woodlands in the region [24] has shown that atypids are relatively common (and unnoticed) across Delaware and Philadelphia counties and into adjacent areas of Montgomery and Chester counties (Fig 6). The polygon in the map shows the extent of vertical purse-web sightings as an estimate of the species’ distribution, including all previously known and verified *A*. *karschi* sites (as *A*. *snetsingeri*), but the webs alone are not diagnostic [7,25]. It is possible that vertical-web building *S*. *rufipes* or horizontal-web building *S*. *niger* [64,65] also occur in that area but are as yet undetected, and those species may be documented as purse-web sites are revisited to make species determinations. Atypid field surveys can utilize external morphological features to identify wandering males and excavated females [71]. Mature males of *A*. *karschi* are distinguished from northern *Sphodros* species (*S*. *niger*, *S*. *rufipes*, *S*. *atlanticus*) by their color, small palps and a ridge around the sternum, while females and immatures are distinguished from *Sphodros* by their sternum sigilla pattern and the posterior lateral spinnerets [PLS, 25]. In particular, *A*. *karschi* has distinctly four-segmented PLS, whereas the northern *Sphodros* species have only three segments.

Interestingly, *Sphodros* purse-web spiders have been reported in Pennsylvania and adjacent states [25], but not in the same areas as *A*. *karschi*. This is unsurprising because atypids and their webs are rarely noticed or reported even when they are locally abundant [24,71], and while perhaps provocative, those observations are not evidence of displacement of any local species by the introduction of *A*. *karschi*. *Sphodros* may yet be identified within the estimated *A*. *karschi* range. Of historic interest, the first "red-legged" purse-web spider reported from North America was in 1829, a male taken "near Philadelphia" and sent to France to later become the holotype specimen for *Sphodros rufipes* [25]. A more precise location was not given and since that time *S*. *rufipes* has not been reported in the Philadelphia region of Pennsylvania, with the nearest record in coastal New Jersey. Sightings of wandering *Sphodros* males near southeastern Pennsylvania and reported in iNaturalist [72] indicate that: 1) *S*. *rufipes* occurs nearby in coastal regions of Maryland and New Jersey south and east of the Philadelphia area, and northward into New England; 2) *S*. *niger* was found west and north of Philadelphia within Pennsylvania and nearby in the states of New Jersey, Delaware and Maryland; and, 3) *S*. *atlanticus* is reported south of the *A*. *karschi* area in coastal Delaware and Maryland. More research is needed on North American atypid spiders to clarify their distributions, habitat and vegetation associations, prey preferences, possible interactions and impacts on their local ecology. Those data will also facilitate determining whether the distributions of *Sphodros* and *A*. *karschi* change in the future in response to regional land-use practices, local habitat destruction and development, or climate change.

It is unlikely that the source and timing of *A*. *karschi*’s introduction to Pennsylvania will ever be determined. The species has a broad native range in East Asia extending from China and Taiwan to Japan [14], and it was recently also reported in Korea [69]. The Philadelphia region (including Delaware County) has had a 300 year history of trade with East Asia that may have included countless opportunities for accidental importation of a soil-associated spider among potted plants. Indeed, Nentwig [73] suggests that spiders introduced with potted plants have higher establishment rates relative to those introduced by other means. In the 1700s and 1800s Philadelphia was the center of American botany and horticulture and many plants from around the world, including Asia, were actively collected, imported and traded for exhibition and cultivation in public and private gardens [74,75]. Many of the region’s great gardens and arboreta of that era still exist to some extent [76], including Tyler Arboretum (visited in this study) and Bartram’s Garden in west Philadelphia, the home of noted American botanists John Bartram and his son William [77,78]. William Bartram’s contemporary in the late 1700s, William Hamilton, built his estate “The Woodlands” overlooking Philadelphia’s Schuylkill River and his gardens and greenhouse boasted of having every rare plant he’d ever heard of from around the world [79,80]. In 1784, after the American Revolution, direct shipping trade began between Philadelphia and China and at its peak represented about a third of all US trade with China [81]. A very significant Asian botanical importation event occurred later, in 1926, when the Japanese government presented 1,600 flowering trees to the City of Philadelphia to celebrate the 150^th^ anniversary of American independence [82]. Regarding introductions of other soil-associated invertebrates, Asian jumping worms (*Amynthas* and *Metaphire* spp.) were presumably brought to the US in the 1800s in the soil of potted plants, and recent studies have shown that they displace native worms and are changing the soil where they occur [83]. Coincidentally, nonnative jumping worms are present at many *A*. *karschi* sites in Pennsylvania [Tessler, personal observations].

## Conclusion

Many spider species have been accidentally introduced by humans to a new continent and became established [73], nearly all from the phylogenetically recent infraorder Araneomorphae. Within the more basal mygalomorphs, the Mexican redrump tarantula (Theraphosidae) native to Mexico and Central America has become established in Florida USA [84]. Presumably escaped from the pet trade, these tarantulas dig burrows and appear to be restricted to a small area with climate and habitat features similar to its native range. In this study we show that *Atypus snetsingeri* in Pennsylvania is genetically conspecific with *Atypus karschi* native to East Asia. The species appears to have been introduced by humans to Pennsylvania, probably in association with potted plants, and is now naturalized and locally common within a limited range that includes urban and forested areas. It is unlikely that the source or timing of the introduction can be determined in a region renowned for its colonial-era horticulturalists, elaborate international gardens, and long history of shipping trade with East Asia. This is the first case of an introduced species of Atypoidea from the infraorder Mygalomorphae.

## Supporting information

S1 File Characteristics of the studied sites of *A*. *karschi* in Delaware County, Pennsylvania, USA in November 2013: Location, date of visit, site orientation and slope, soil type and penetrability, and the cover and composition of the vegetation strata. The abundance of plant species is characterized using standardized ranks (Braun-Blanquet 1932).
(DOC)

S2 File Carapace length, web length and number of juveniles with adult females of *Atypus karschi*, Pennsylvania, USA (n = 18). NA–the web was destroyed while excavating and could not be measured.
(DOC)

## References

[1] Wheeler WC, Coddington JA, Crowley LM, Dimitrov D, Goloboff PA, Griswold CE, et al. The spider tree of life: phylogeny of Araneae based on target-gene analyses from an extensive taxon sampling. Cladistics. 2017;33(6): 574–616. doi: 10.1111/cla.12182 34724759

[2] Enock F. The life-history of *Atypus piceus*, Sulz. Trans R Entomol Soc Lond. 1885;33(4): 389–420.

[3] McCook HC. Nesting habits of the American purseweb spider. Proc Acad Nat. 1888;40: 203–220. Available from: https://www.biodiversitylibrary.org/bibliography/6885.

[4] Poteat WL. A tube-building spider. Notes on the architectural and feeding habits of Atypus niger Hentz. J Elisha Mitchell Sci Soc. 1890;6(2): 132–47.

[5] Platnick NI. Spiders of the world: A natural history. London: Princeton University Press; 2020.

[6] Bristowe WS. World of spiders. Collins; 1958.

[7] Schwendinger PJ. A synopsis of the genus Atypus (Araneae, Atypidae). Zool Scr. 1990;19(3): 353–66.

[8] Řezáč M, Heneberg P. Conservation status of the only representative of infraorder Mygalomorphae (Araneae) in cultivated regions of central Europe. J Insect Conserv. 2014;18(4): 523–37.

[9] Coyle FA. Aerial Dispersal by mygalomorph spiderlings (Araneae, Mygalomorphae). J Arachnol. 1983;11(2): 283–6.

[10] Coyle FA, Greenstone MH, Hultsch A-L, Morgan CE. Ballooning mygalomorphs: Estimates of the masses of Sphodros and Ummidia ballooners (Araneae: Atypidae, Ctenizidae). J Arachnol. 1985;13(3): 291–6.

[11] Buzatto BA, Haeusler L, Tamang N. Trapped indoors? Long-distance dispersal in mygalomorph spiders and its effect on species ranges. J Comparative Physiology A. 2021; 207: 279–292. doi: 10.1007/s00359-020-01459-x 33515318

[12] Nentwig W, Blick T, Bosmans R, Gloor D, Hänggi A, Kropf C. Spiders of Europe. Version 5; 2021 [cited 20 May 2021]. Database [Internet]. Available from: https://www.araneae.nmbe.ch

[13] Zhu MS, Zhang F, Song DX, Qu P. A revision of the genus Atypus in China (Araneae: Atypidae). Zootaxa. 2006; 1118: 1–42.

[14] World Spider Catalog. World Spider Catalog; 2021 [cited 30 Mar 2021]. Natural History Museum Bern. [Internet]. Available from: http://wsc.nmbe.ch.

[15] Dippenaar-Schoeman AS, Jocqué R. African spiders: an identification manual. Pretoria: ARC-Plant Protection Research Institute; 1997.

[16] Řezáč M, Řezáčová V, Pekár S. The distribution of purse-web Atypus spiders (Araneae: Mygalomorphae) in central Europe is constrained by microclimatic continentality and soil compactness. J Biogeogr. 2007;34(6): 1016–27.

[17] Řezáč M, Tošner J, Heneberg P. Habitat selection by threatened burrowing spiders (Araneae: Atypidae, Eresidae) of central Europe: evidence base for conservation management. J Insect Conserv. 2018 Feb 1;22(1): 135–49.

[18] National Center for Biotechnology Information (NCBI). Query results for Atypidae genome data; 2021 [cited 23 Oct 2021] Database [Internet]. Available from: https://www.ncbi.nlm.nih.gov/nuccore/?term=atypidae.

[19] Král J, Kořínková T, Forman M, Krkavcová L. Insights into the meiotic behavior and evolution of multiple sex chromosome systems in spiders. Cytogenet Genome Res. 2011;133(1): 43–66. doi: 10.1159/000323497 21282941

[20] Řezáč M, Král J, Musilová J, Pekár S. Unusual karyotype diversity in the European spiders of the genus Atypus (Araneae: Atypidae). Hereditas. 2006;143(2006): 123–9. doi: 10.1111/j.2006.0018-0661.01949.x 17362345

[21] Suzuki S. Cytological studies in spiders. III. Studies on the chromosomes of fifty-seven species of spiders belonging to seventeen families, with general considerations on chromosomal evolution. J Sci Hiroshima Univ(B). 1954;15: 23–136.

[22] Král J, Kořínková T, Krkavcová L, Musilová J, Forman M, Ávila Herrera IM, et al. Evolution of karyotype, sex chromosomes, and meiosis in mygalomorph spiders (Araneae: Mygalomorphae). Biol J Linn Soc. 2013;109(2): 377–408.

[23] Sarno PA. New species of Atypus. Entomol News Philadelphia. 1973; 37–51.

[24] Tessler S. Map the spider; 2022 [cited 22 Apr 2022]. Available from: http://www.mapthespider.com/.

[25] Gertsch WJ, Platnick NI. A revision of the American spiders of the family Atypidae (Araneae, Mygalomorphae). Am Mus Novit; no. 2704; 1980. Available from: https://digitallibrary.amnh.org/handle/2246/5390.

[26] Raven RJ. The spider infraorder Mygalomorphae (Araneae): cladistics and systematics. Bull Am Mus Nat. 1985;182. Available from: https://digitallibrary.amnh.org/handle/2246/955.

[27] Řezáč M, Arnedo MA, Opatova V, Musilová J, Řezáčová V, Král J. Revision and insights into the speciation mode of the spider Dysdera erythrina species-complex (Araneae:Dysderidae): sibling species with sympatric distributions. Invertebr Syst. 2018;32: 10–54.

[28] Ávila Herrera IM, Král J, Pastuchová M, Forman M, Musilová J, Kořínková T, et al. Evolutionary pattern of karyotypes and meiosis in pholcid spiders (Araneae: Pholcidae): implications for reconstructing chromosome evolution of araneomorph spiders. BMC Ecol Evol. 2021; 21(1): 75. doi: 10.1186/s12862-021-01750-8 33941079 PMC8091558

[29] Srba M, Heneberg P. Nesting habitat segregation between closely related terricolous sphecid species (Hymenoptera:Spheciformes): key role of soil physical characteristics. J Insect Conserv. 2012;16(4): 557–70.

[30] R Core Team. R: A language and environment for statistical computing [Internet]. Vienna, Austria: R Foundation for Statistical Computing; 2019. Available from: http://www.r-project.org/.

[31] Schneider CA, Rasband WS, Eliceiri KW. NIH Image to ImageJ: 25 years of image analysis. Nature methods. 2012;9(7): 671–5. doi: 10.1038/nmeth.2089 22930834 PMC5554542

[32] Levan A, Fredga K, Sandberg AA. Nomenclature for centromeric position on chromosomes. Hereditas. 1964;52(2): 201–20.

[33] Král J, Kováč L, Št’áhlavský F, Lonský P, L’uptáčik P. The first karyotype study in palpigrades, a primitive order of arachnids (Arachnida: Palpigradi). Genetica. 2008;134(1): 79–87. doi: 10.1007/s10709-007-9221-y 18030430

[34] Forman M, Nguyen P, Hula V, Král J. Sex chromosome pairing and extensive NOR polymorphism in Wadicosa fidelis (Araneae: Lycosidae).Comp Genome Res. 2013;141(1): 43–9. 10.1159/000351041 23711575

[35] Heneberg P, Řezáč M. Two Trichosporon species isolated from Central-European mygalomorph spiders (Araneae: Mygalomorphae). Antonie van Leeuwenhoek. 2013 Apr 1;103(4):713–21. doi: 10.1007/s10482-012-9853-5 23180375

[36] White TJ, Bruns T, Lee S, Taylor J. Amplification and direct sequencing of fungal ribosomal RNA genes for phylogenetics. PCR protocols: a guide to methods and applications. 1990;18(1): 315–22.

[37] Folmer O, Black M, Wr H, Lutz R, Vrijenhoek R. DNA primers for amplification of mitochondrial cytochrome C oxidase subunit I from diverse metazoan invertebrates. Mol Mar Bio Biotechnol. 1994;3: 294–9. 7881515

[38] Bidegaray‐Batista L, Macías‐Hernández N, Oromí P, Arnedo MA. Living on the edge: demographic and phylogeographical patterns in the woodlouse-hunter spider *Dysdera lancerotensis* Simon, 1907 on the eastern volcanic ridge of the Canary Islands. Mol Ecol. 2007;16(15): 3198–214. doi: 10.1111/j.1365-294X.2007.03351.x 17651197

[39] Madeira F. Park Y. Lee J, Buso N, Gur T, Madhusoodanan N, et al. The EMBL-EBI search and sequence analysis tools APIs in 2019. Nucleic Acids Res. 2019;47: W636–W641. doi: 10.1093/nar/gkz268 30976793 PMC6602479

[40] National Center for Biotechnology Information (NCBI). Query results for Atypus genome data [Internet]. 2021 [cited 2021 Jul 1]. Available from: https://www.ncbi.nlm.nih.gov/nuccore/?term=Atypus%20[Organism].

[41] Hedin M, Derkarabetian S, Alfaro A, Ramírez MJ, Bond JE. Phylogenomic analysis and revised classification of atypoid mygalomorph spiders (Araneae, Mygalomorphae), with notes on arachnid ultraconserved element loci. PeerJ. 2019;7: e6864. doi: 10.7717/peerj.6864 31110925 PMC6501763

[42] Hedin M, Bond JE. Molecular phylogenetics of the spider infraorder Mygalomorphae using nuclear rRNA genes (18S and 28S): conflict and agreement with the current system of classification. Mol Phylogenet Evol. 2006;41(2): 454–71. doi: 10.1016/j.ympev.2006.05.017 16815045

[43] Bond JE, Hendrixson BE, Hamilton CA, Hedin M. A reconsideration of the classification of the spider infraorder Mygalomorphae (Arachnida: Araneae) based on three nuclear genes and morphology. PLOS One. 2012;7(6): e38753. doi: 10.1371/journal.pone.0038753 22723885 PMC3378619

[44] Ayoub NA, Garb JE, Hedin M, Hayashi CY. Utility of the nuclear protein-coding gene, elongation factor-1 gamma (EF-1γ), for spider systematics, emphasizing family level relationships of tarantulas and their kin (Araneae: Mygalomorphae). Mol Phylogenet Evol. 2007;42(2): 394–409. doi: 10.1016/j.ympev.2006.07.018 16971146

[45] He A, Guo J, Peng H, Huang Z, Liu J, Xu X. The complete mitochondrial genome of Atypus karschi (Araneae, Atypidae) with phylogenetic consideration. Mitochondrial DNA Part B. 2021;6(9): 2523–5. doi: 10.1080/23802359.2021.1959443 34377816 PMC8330730

[46] Araujo D, Schneider MC, Paula-Neto E, Cella DM. The World spider cytogenetic database [Internet]. 2020 [cited 30 Apr 2020]. Available from: http://www.arthropodacytogenetics.bio.br/spiderdatabase/

[47] Kořínková T, Král J. Karyotypes, sex chromosomes, and meiotic division in spiders. In: Spider ecophysiology. Springer; 2013. p. 159–71.

[48] Sember A, Pappová M, Forman M, Nguyen P, Marec F, Dalíková M, et al. Patterns of sex chromosome differentiation in spiders: Insights from comparative genomic hybridisation. Genes. 2020;11(8): 849. doi: 10.3390/genes11080849 32722348 PMC7466014

[49] Clark MS, Wall WJ. Chromosomes: the complex code. Chapman & Hall Ltd; 1996.

[50] Miller DA, Dev VG, Tantravahi R, Miller OJ. Suppression of human nucleolus organizer activity in mouse-human somatic hybrid cells. ExpCell Res. 1976;101(2): 235–43. doi: 10.1016/0014-4827(76)90373-6 61125

[51] Fujiwara A, Abe S, Yamaha E, Yamazaki F, Yoshida MC. Chromosomal localization and heterochromatin association of ribosomal RNA gene loci and silver-stained nucleolar organizer regions in salmonid fishes. Chromosome Res. 1998;6(6): 463–71. doi: 10.1023/a:1009200428369 9865785

[52] Guetg C, Santoro R. Formation of nuclear heterochromatin. Epigenetics. 2012;7(8): 811–4. doi: 10.4161/epi.21072 22735386 PMC3427276

[53] Carmo-Fonseca M, Mendes-Soares L, Campos I. To be or not to be in the nucleolus. Nat Cell Biol. 2000;2(6): E107–12. doi: 10.1038/35014078 10854340

[54] Miyashita K. Postembryonic development and life cycle of Atypus karschi DOENITZ (Araneae: Atypidae). Acta Arachnol. 1992;10: 177–186.

[55] iNaturalist. Images of Atypus karschi. [Internet]. 2021 [cited 28 Jul 2021]. Available from: https://www.inaturalist.org/observations?verifiable=true&taxon_id=360542.

[56] British Arachnology Society. Spider and harvestman recording scheme website. Summary for Atypus affinis (Araneae). [Internet]. 2021 [cited 29 Jul 2021 ]. Available from: http://srs.britishspiders.org.uk/portal.php/p/Summary/s/Atypus+affinis.

[57] Aoki T. On the life cycle of Jigumo (Atypus karschi Dön.) in Wakajama city. Nanki-Seibutsu Suppl. 1983;25: 43–8.

[58] Yaginuma T. Spiders of Japan in colour (enl, rev. ed.). Hoikusha Publishing Co.; 1978.

[59] Zhu M-S, Zhang F, Song D, Qu P. A revision of the genus Atypus in China (Araneae: Atypidae). Zootaxa. 2006;1118(1): 1–42.

[60] Bösenberg W, Strand E. Japanische spinnen. Abh. Senckenb. Naturforsch. Ges. 1906; 30: 93–422.

[61] Dönitz W. Uber die Lebensweise Zweier Vogelspinnen aus Japan. Sitzungsber. Gesell. Naturf. Freunde Berlin. 1887;8–10.

[62] Schwendinger PJ. On the genus Atypus (Araneae: Atypidae) in northern Thailand. Bull Br Arachnol Soc. 1989;8(3): 89–96.

[63] Beatty JA. Web structure and burrow location of Sphodros niger (Hentz) (Araneae: Atypidae). J Arachnol. 14: 130–132. Available from: https://www.americanarachnology.org/journal-joa/joa-all-volumes/detail/article/download/JoA_v14_p130_grey.pdf.

[64] Edwards RL, Edwards EH. Observations on the natural history of a New England population of Sphodros niger (Araneae, Atypidae). J Arachnol. 1990;29–34.

[65] Mckenna-Foster A, Draney ML, Beaton C. An unusually dense population of Sphodros rufipes (Mygalomorphae: Atypidae) at the edge of its range on Tuckernuck Island, Massachusetts. J Arachnol. 2011;39(1): 171–3.

[66] Yih D. Land bridge travelers of the Tertiary: the Eastern Asian–Eastern North American floristic disjunction. Arnoldia. 2012; 69: 14–23.

[67] Sanmartín I, Enghoff H, Ronquist F. Patterns of animal dispersal, vicariance and diversification in the Holarctic. Biol J Linnean Soc. 2001; 73: 345–390.

[68] Saitō S. On the spiders from Tohoku (northernmost part of the main island), Japan. Saito Ho-on Kai Museum Research Bulletin. 1939;18: 1–91.

[69] Kim J, Cho Y. Unrecorded Korean Atypus karschii Doenitz (Atypidae Thorell, Genus Atypus). Korean Arachnology. 2019;35 (1–2): 1–4.

[70] Katsura K. A note on a ballooning of Atypus karschi. Atypus. 1975;64: 6.

[71] Moler PE, Stevenson DJ, Mansell BW, Mays JD, Lee CW. Distribution and natural history of purseweb spiders, Sphodros spp. (Araneae: Mygalomorphae: Atypidae), in Florida, Georgia, and Alabama. Southeast Nat. 2020;19(4): 663–72.

[72] iNaturalist. Map of the Genus Sphodros. [Internet]. 2021 [cited 21 Jul 2021]. Available from: https://www.inaturalist.org/observations?place_id=any&subview=map&taxon_id=61444.

[73] Nentwig W. Introduction, establishment rate, pathways and impact of spiders alien to Europe. Biol Invasions. 2015;17(9): 2757–78.

[74] Chesney S. Encyclopedia of Greater Philadelphia. Botany. [Internet]. 2017 [cited 14 Dec 2020]. Available from: https://philadelphiaencyclopedia.org/archive/botany/.

[75] Sarudy B. Colonial and early American gardens: The greenhouse in early America [Internet]. 2020 [cited 24 Aug 2021]. Available from: https://americangardenhistory.blogspot.com/2018/12/the-greenhouse-conservatory-in-early.html.

[76] Klein WM. Gardens of Philadelphia & the Delaware Valley. Temple University Press; 1995.

[77] Rehder A. On the history of the introduction of woody plants into North America. Arnoldia 6: 13–23 [Internet]. 1946 [cited 21 Jun 2021]. Available from: http://arnoldia.arboretum.harvard.edu/pdf/articles/1946-6—on-the-history-of-the-introduction-of-woody-plants-into-north-america.pdf.

[78] O’Malley T. The evidence of American garden history. History of early American landscape design [Internet]. 2021 [cited 24 Aug 2021]. Available from: https://heald.nga.gov/mediawiki/index.php?title=The_Evidence_of_American_Garden_History&oldid=40049.

[79] Stetson SP. William Hamilton and his" Woodlands". Pa. Mag. Hist. Biogr. 1949;73(1): 26–33.

[80] Madsen K. To make his country smile: William Hamilton’s woodlands. Arnoldia. 1989;49(2): 14–24.

[81] Norwood D. The encyclopedia of Greater Philadelphia. China Trade. [Internet]. 2016 [cited 15 Jun 2021]. Available from: https://philadelphiaencyclopedia.org/archive/china-trade/.

[82] Japan Society of Greater Philadelphia. Shofuso. History [Internet]. 2021 [cited 15 Jul 2021]. Available from: https://japanphilly.org/programs/festivals/subaru-cherry-blossom-festival/about/history./

[83] Qiu J, Turner MG. Effects of non-native Asian earthworm invasion on temperate forest and prairie soils in the Midwestern US. Biol Invasions. 2017;19(1): 73–88.

[84] Edwards GB, Hibbard K. The Mexican Redrump, Brachypelma vagans (Araneae: Theraphosidae), an exotic tarantula established in Florida. Entomology Circular No. 394, May/June 1999, 2pp. [Internet]. 1999 [cited 19 Jul 2021]. Available from: https://www.fdacs.gov/content/download/10780/file/ent394.pdf.

