# Supplementary material for: Atypus karschi Dönitz, 1887 (Araneae: Atypidae): An Asian purse-web spider established in Pennsylvania, USA
Source: PLoS One. 2022 Jul 7;17(7):e0261695. doi: 10.1371/journal.pone.0261695 (PMC9262232; doi:10.1371/journal.pone.0261695)
Supplement: S1 File — The abundance of plant species is characterized using standardized ranks (Braun-Blanquet 1932). (DOC) [file pone.0261695.s001.doc]

**Supporting Information 1.** Characteristics of the studied sites of *Atypus karschi* in Delaware County, Pennsylvania, USA in November 2013: location, date of visit, site orientation and slope, soil type and penetrability, and the cover and composition of the vegetation strata. The abundance of plant species is characterized using standardized ranks (Braun-Blanquet 1932).

| site | Swedish Cabin | Lansdowne, Essex Ave | Naylor Run Park | Smedley Park | Smedley Park | Tyler Arboretum | Tyler Arboretum | Tyler Arboretum |
| --- | --- | --- | --- | --- | --- | --- | --- | --- |
| GPS coordinates | 39.93698, -75.30093 | 39.9444, -75.27606 | 39.957009, -75.27949 | 39.917311, -75.359051 | 39.917311, -75.359051 | 39.929369, -75.435086 | 39.928783, -75.434941 | 39.941767, -75.429050 |
| date | 5 Nov 2013 | 5 Nov 2013 | 5 Nov 2013 | 5 Nov 2013 | 5 Nov 2013 | 6 Nov 2013 | 9 Nov 2013 | 9 Nov 2013 |
| habitat | bottom of the valley | city suburb | shallow valley | rocky valley | rocky valley | fallow field | field-adjacent beech forest | beech forest |
| orientation | 95° | none | 195° | 340° | 260–270° | 310° | - | 180° |
| slope | 5° | 0° | 15° | 25° | 40° | 10° | 0° | 35° |
| Soil type | Fluvisol | Synantropic | Sandy | Lower layer yellow | Lower layer yellow | Topsoil | Grey | Yellow |
| Soil penetrability (kg/cm^2^) | 1-5 |  | 0.5–2 | 0.5–1.75 | 2–3 | 2.25–3.25 | 1.5–2.25 | 1.25–2 |
| moss cover (%) | 0 | 0 | 0 | 0 | 0 | 0 | 0 | 0 |
| herb cover (%) | 20 | 0 | 5 | 10 | 30 | 90 | 0 | 20 |
| bush cover (%) | 80 | 100 | 20 | 40 | 20 | 5 | 30 | 10 |
| tree cover (%) | 50 | 0 | 90 | 60 | 80 | 0 | 60 | 80 |
| **Herb cover** |  |  |  |  |  |  |  |  |
| *Carex* sp. 1 | 1 |  |  | 1 | 2 |  |  | + |
| *Carex* sp. 2 |  |  |  |  | + |  |  |  |
| *Hieracium* cf. *venosum* |  |  |  |  | + |  |  |  |
| *Brachypodium* sp. |  |  |  | 1 |  |  |  |  |
| *Polystichum* sp. |  |  |  |  | + |  |  |  |
| *Solidago* sp. |  |  |  |  | 1 | 4 |  |  |
| *Geum* sp. |  |  |  |  |  | r |  |  |
| *Rubus* sp. | 1 |  |  |  |  | 1 |  |  |
| *Dactylis* sp. |  |  |  |  |  | + |  |  |
| *Lonicera* sp. |  |  |  |  |  | 1 |  | 2 |
| *Cirsium* sp. |  |  |  |  |  | r |  |  |
| *Linaria* sp. |  |  |  |  |  | r |  |  |
| *Daucus carota* |  |  |  |  |  | r |  |  |
| *Medicago* sp. |  |  |  |  |  | r |  |  |
| *Allium* sp. |  |  | + |  |  |  |  | + |
| *Hedera* sp. | 2 | 3 | + |  |  |  |  |  |
| *Luzula* sp. |  |  |  |  | + |  |  |  |
| *Fragaria* sp. | r |  |  |  |  |  |  |  |
| *Alliaria petiolata* | + |  |  |  |  |  |  |  |
| *Desmodium* sp. |  |  |  |  |  | 1 |  |  |
| **Bush cover** |  |  |  |  |  |  |  |  |
| *Fagus grandifolia* |  |  | 2 | 2 | 2 |  |  | 2 |
| *Corylus* sp. |  |  |  | 1 |  |  |  |  |
| *Rosa* sp. | 2 |  | + |  |  | + |  | + |
| *Ulmus* sp. |  |  |  |  |  | r |  |  |
| *Taxus* sp. |  | 5 |  |  |  |  |  |  |
| *Viburnum* sp. |  |  |  |  | + |  |  |  |
| *Lonicera* sp. | 2 |  |  |  |  |  |  |  |
| *Ligustrum* sp. | 2 |  |  |  |  |  |  | + |
| **Tree cover** |  |  |  |  |  |  |  |  |
| *Acer* cf. *saccharum* | 2 |  | 5 | 1 |  |  |  |  |
| *Populus* cf. *grandidentata* | 1 |  |  | 1 |  |  |  |  |
| *Fraxinus* sp. | 1 |  |  |  |  |  |  |  |
| *Quercus rubra* | 2 |  | 1 | 3 |  |  |  |  |
| *Quercus* cf. *alba* |  |  | 1 |  | 4 |  | 1 | 1 |
| *Carpinus caroliniana* |  |  |  | 1 |  |  |  |  |
| *Liliodendron tulipifera* |  |  | 1 | + | 1 |  |  | 1 |
| *Juglans* sp. | + |  |  |  |  |  |  |  |
| *Fagus grandifolia* |  |  |  |  |  |  | 4 | 4 |
