# Supplementary material for: Atypus karschi Dönitz, 1887 (Araneae: Atypidae): An Asian purse-web spider established in Pennsylvania, USA
Source: PLoS One. 2022 Jul 7;17(7):e0261695. doi: 10.1371/journal.pone.0261695 (PMC9262232; doi:10.1371/journal.pone.0261695)
Supplement: S2 File — NA–the web was destroyed while excavating and could not be measured. (DOC) [file pone.0261695.s002.doc]

**Supporting Information 2.** Carapace length (smallest to largest), number of juveniles present and web length for excavated purse-webs of adult female *Atypus karschi*, Pennsylvania, USA, in November 2013 (n = 18). NA – the web was destroyed while excavating and could not be measured. * - incomplete count or measurement and not used for statistics.

| **Carapace length (mm)** | **No. of juveniles present** | **Length of below-ground web (cm)** | **Length of above-ground web (cm)** | **Total web length (cm)** |
| --- | --- | --- | --- | --- |
| 5.0 | 123 | 9 | 7.5 | 16.5 |
| 5.1 | 70 | NA | NA |  |
| 5.2 | 0 | 10 | 8 | 18 |
| 5.2 | 106 | 8 | 9 | 17 |
| 5.4 | 152 | 8 | 10.5 | 18.5 |
| 5.4 | 129 | NA | NA |  |
| 5.5 | 116 | 8 | 9.5 | 17.5 |
| 5.5 | 0 | NA | NA |  |
| 5.6 | 27* | 8 | 9 | 17 |
| 5.8 | 0 | 9 | 5 | 14 |
| 5.9 | 79 | 10 | 10 | 20 |
| 5.9 | 128 | 8.5 | 6 | 14.5 |
| 6.0 | 0 | 7 | 6 | 13 |
| 6.0 | 0 | 16 | 13 | 29 |
| 6.1 | 201 | 8 | 7 | 15 |
| 6.2 | 0 | 8 | 13 | 21 |
| 6.2 | 109 | 6* | 6 |  |
| 6.2 | 0 | NA | NA |  |
